# Pulvinar responsive neurostimulation for posterior quadrant epilepsy: an illustrative case with connectome projection

**DOI:** 10.1007/s10143-025-03966-4

**Published:** 2025-12-08

**Authors:** Bernardo Assumpcao de Monaco, Alejandro N. Santos, Joacir Graciolli Cordeiro, Eduardo Joaquim Lopes Alho, Guilherme Santos Piedade, Bashar Dawoud, Ahmed Doomi, Ryan O’Boyle, Andres M. Kanner, Naymee Velez-Ruiz, Jonathan R. Jagid

**Affiliations:** 1 Neurosurgery - Camden Clark Medical Center, West Virginia Universtiy, Parkersburg, WV USA; 2 Clinica de Dor e Funcional, Sao Paulo, SP Brazil; 3 Department of Neurosurgery, University of Miami, Miami, FL USA; 4 Department of Neurosurgery, University of Pittsburgh Medical Center, Altoona, PA USA; 5 Neurology Department, University of Miami, Miami, FL USA

**Keywords:** Drug resistant epilepsy, Deep brain stimulation, Responsive neurostimulation, Neuromodulation, Pulvinar, Tractography

## Abstract

Objective To present the rationale for targeting the thalamic pulvinar nucleus with Responsive Neurostimulation (RNS) in refractory epilepsy involving the posterior quadrant, supported by connectome-based analysis and a rare illustrative case of a successful pulvinar implantation. Methods A connectome-based analysis of the pulvinar nucleus was conducted using normative diffusion imaging data from 1,065 subjects to map structural connectivity with cortical and subcortical regions implicated in seizure propagation. Insights from literature were combined with a detailed clinical case in which RNS electrodes were implanted in the pulvinar nucleus and posterior cortical regions after extensive presurgical evaluation. Results Connectome analysis demonstrated extensive pulvinar connectivity with seizure-prone regions, including mesial temporal, parietal, and occipital cortices, supporting its role in modulating corticothalamic activity. The illustrative patient, with drug-resistant posterior quadrant epilepsy not amenable to resection, underwent pulvinar and cortical RNS lead placement. At 18-month follow-up, clinical seizures were reduced to only two episodes, both associated with sleep deprivation, with ECoG evidence of aborted interictal runs by therapy. Conclusion This report underscores the thalamic pulvinar nucleus as a promising yet rarely targeted site for neuromodulation in posterior quadrant epilepsy. The combination of connectome-based targeting and successful long-term clinical outcome supports further exploration of pulvinar RNS as a treatment option for complex epilepsy cases where conventional targets may be suboptimal.

## Introduction

Drug resistant epilepsy (DRE), affecting approximately 30–40% of patients with epilepsy, presents a formidable challenge in clinical practice [1]. While resective and ablative surgical interventions have demonstrated efficacy [2], their success is heavily dependent on a single epileptogenic area and/or the absence of involvement of eloquent cortical regions within it[3]. For cases where surgical resection is not feasible, neuromodulation therapies have emerged as viable alternatives. These include vagus nerve stimulation (VNS), deep brain stimulation (DBS), and responsive neurostimulation system (RNS) systems, all of which have shown promise in reducing seizure frequency and improving patient outcomes [4]. Among these neuromodulation therapies, RNS and DBS targeting the anterior nucleus of the thalamus have received considerable attention and validation [5, 6]. However, the therapeutic potential of other targets remain less explored, despite their involvement in epileptogenesis [5]. Among these, the pulvinar nucleus is of particular interest given its extensive bidirectional connectivity with parietal, occipital, and mesial temporal cortices, making it a strategic hub for modulating seizure propagation in posterior quadrant epilepsies [4, 7–9]. Yet, clinical use of the pulvinar as a neuromodulation target remains limited to a few small series and isolated reports, highlighting the need for detailed clinical documentation and outcome data. Advances in circuit-mapping techniques, including optogenetics, further support investigating such network hubs for epilepsy treatment [10]. In this context, we propose the pulvinar nucleus of the thalamus as a compelling target for neuromodulation, given its extensive connections with both mesial temporal and neocortical regions in posterior head regions involved in seizure genesis [4, 7–9]. Preliminary evidence suggests that pulvinar stimulation may be effective in seizure control, even in pediatric populations [6]. Here, we present what to our knowledge is one of the few documented cases of successful long-term RNS therapy targeting the pulvinar nucleus in a patient with posterior quadrant epilepsy, integrated with connectome-based tractography to illustrate the anatomical rationale for this rare target. This combined anatomical–clinical approach underscores the potential role of pulvinar RNS to provide seizure control in otherwise inoperable cases and to expand treatment options for refractory epilepsy originating in posterior regions, warranting broader investigation.

## Methods

### Normative connectome source and processing

We utilized a normative connectome comprising diffusion imaging data from 1,065 subjects available in Diffusion Spectrum Imaging (DSI) Studio [11]. This population-averaged atlas provides a reliable estimation of macroscale structural connectivity.

### Tractography parameters

Tractography was performed using multishell diffusion with b-values of 990, 1985, and 2980 s/mm2 and 90 diffusion directions per shell. Image resolution was 1.25 mm isotropic. The b-table was checked by an automatic quality control routine to ensure its accuracy [12]. Data were reconstructed in MNI space using q-space diffeomorphic reconstruction [13] to obtain the spin distribution function [14] with a sampling length ratio of 1.7 and an output resolution of 1 mm. A deterministic tracking algorithm was employed [15] with the following thresholds: anisotropy = 0.07208, step size = 0.5 mm, angular threshold randomly selected between 15° and 90°, track length between 30 mm and 300 mm, total 30,000 tracks. The restricted diffusion was quantified using restricted diffusion imaging [16]. A deterministic fiber tracking algorithm [15] was used. ROIs were placed on the pulvinar (97, 1.1e + 02, 53) with a volume size of 9.7e + 02 mm cubic. The ROIs were placed also at occipital, parietal, frontal, and temporal lobes. The anisotropy threshold was 0.0720808. The angular threshold was randomly selected from 15 degrees to 90 degrees. The step size was 0.5 mm. Tracks with length shorter than 30 or longer than 300 mm were discarded.

### Anatomical parcellation

**Fig. 1:**
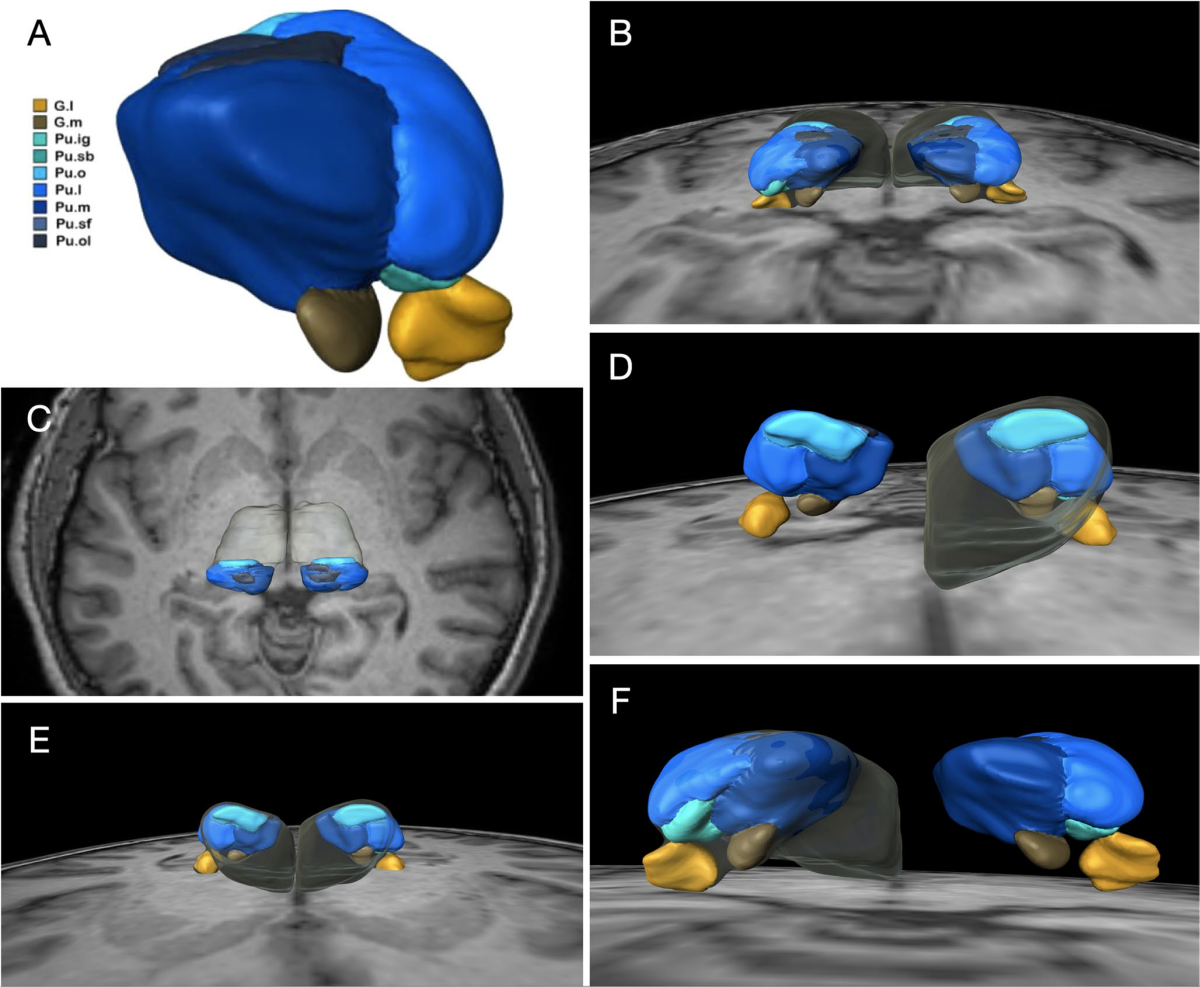
(**A**) Pulvinar spread connections of the left pulvinar with various cortical structures. Notably, there are extensive connections spanning the occipital, parietal, frontal, and temporal lobes. (**B **and **D**) Left pulvinar connections with the ipsilateral occipital lobe. Through the corpus callosum, connections extend to the contralateral occipital lobe, coupled with frontal contralateral connection. (**C**) Right view of the left pulvinar connections to the frontal lobe. (**E**) Temporal connections of the pulvinar and the lemniscal projections in the anterior part of the pulvinar connecting the brainstem and the spinal cord to the somatosensory cortex

The pulvinar was subdivided per Hassler’s thalamic schema [17] (Pu.sf, Pu.o.l, Pu.m, Pu.l, Pu.ig, Pu.sg, Pu.o) using histological sections from the USP-Würzburg Atlas [18] (Fig. 1).

## Results

**Fig. 2:**
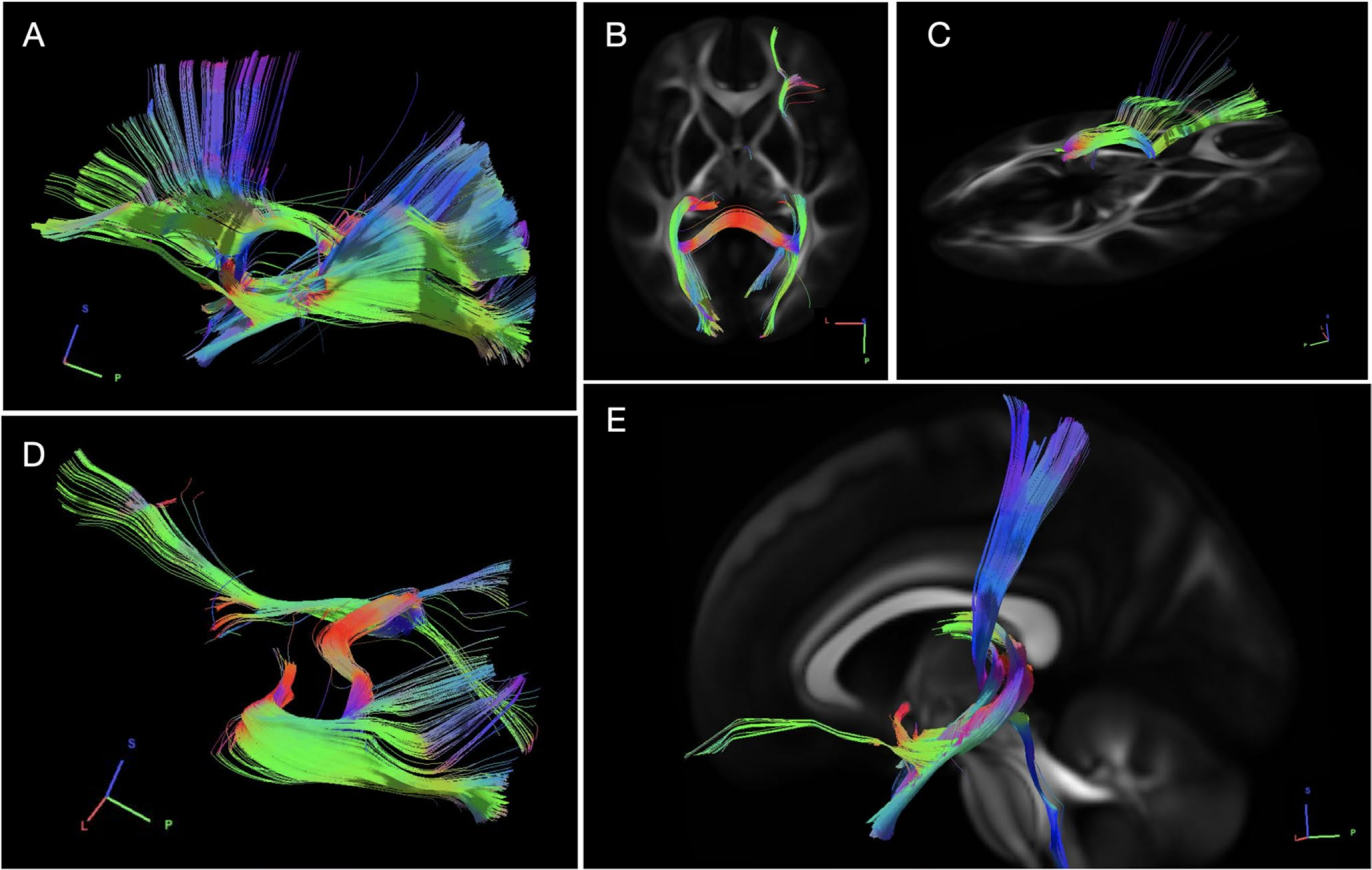
(**A**) Color coding: Pulvinar superficiale (Pu.sf) almost gray; Pulvinar orolaterale (Pu.o.l) dark blue almost black; Pulvinar mediale (Pu.m) darker blue; Pulvinar laterale (Pu.l) medium blue; Pulvinar intergeniculatum (Pu.ig) aqua blue; Pulvinar suprabrachiale (Pu.sg) darker aqua blue; Pulvinar orale (Pu.o) light blue; lateral geniculate nucleus (G. l.) orange; and medial geniculate (G. m.) nucleus brown. (**B**) Posterior view of pulvinar and its subnuclei. (**C**) Superior view. (**D**) Anterolateral view. (**E**) Anterior view. (**F**) Posterolateral view

connectivity with seizure-prone regions, including the mesial temporal, parietal, and occipital cortices (Fig. 2). A total of 30,000 tracts were calculated. This connectivity supports its role in modulating corticothalamic activity. Preliminary clinical observations indicate that Responsive Neurostimulation targeting the pulvinar nucleus may reduce seizure frequency in cases where traditional resective approaches are not feasible.

### Illustrative case

A 39-year-old right-handed female with a history of DRE since the age of 11 presented after multiple failed trials of antiseizure medications (ASM). Her seizure semiology included an aura characterized by a sensation of “head cloudiness” and right-sided facial paresthesia, followed by an epigastric rising sensation, loss of consciousness, and leftward head version. Occasional focal onset to bilateral tonic-clonic (BTC) activity was noted.

At baseline, the patient experienced approximately three to four impaired-awareness seizures per month and two to three focal to bilateral tonic–clonic seizures per year despite treatment with Keppra 750 mg PO BID and Topamax 150 mg PO BID.

Brain MRI showed no focal lesions, cortical dysplasia, or hippocampal sclerosis. Initial EEG recordings demonstrated bifrontal spikes and slow wave activity with a left-sided predominance, along with independent sharp waves in the bitemporal regions. Continuous video EEG (cvEEG) monitoring, particularly during sleep, revealed left posterior quadrant sharp waves that propagated anteriorly to the temporal region, indicating cortical hyperexcitability in the left posterior quadrant. Additional findings included epileptiform discharges in the left mesio-parietal/occipital cortex and, less frequently, in the right mesio-parietal region. A focal seizure originating in the left mesio-parietal/occipital area was observed, which subsequently evolved to bilateral tonic–clonic activity. A PET scan showed no asymmetry in metabolism, and functional MRI (fMRI) confirmed left hemispheric dominance for language. Magnetoencephalography (MEG) highlighted abnormal vectors in the left parietal region.

**Fig. 3:**
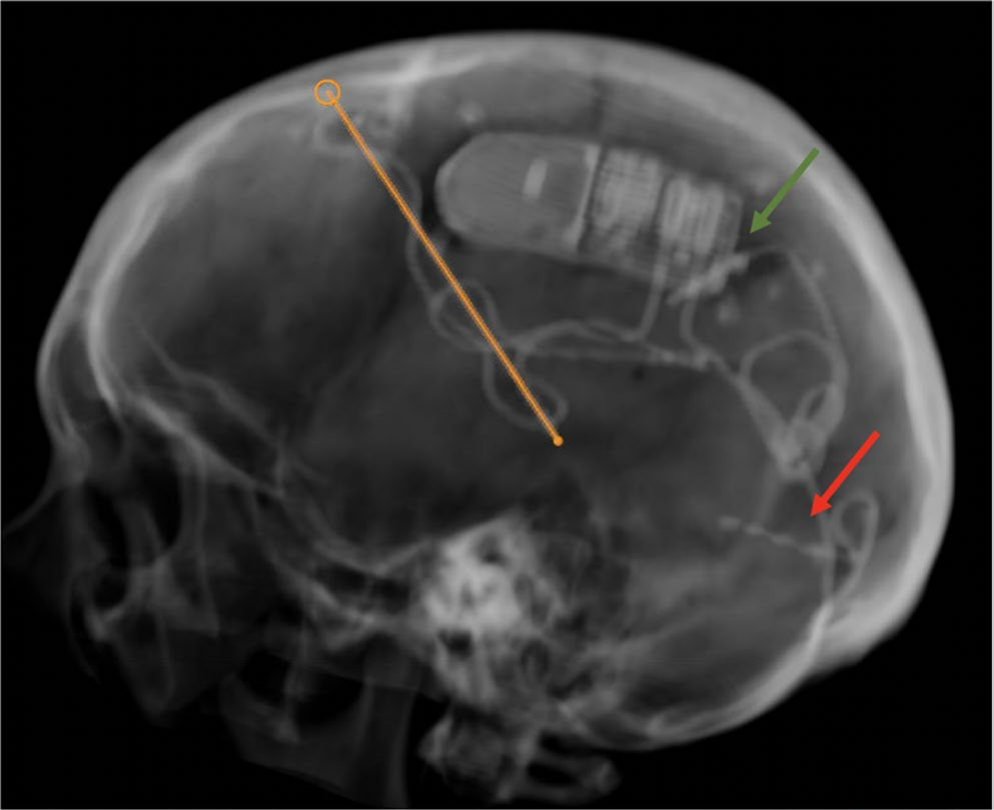
Shows a 3D reconstruction of patient’s skull showing the battery placed in the parietal region connected to 2 leads (the left pulvinar lead traced in orange and the green arrow pointing to left parietal lead). The lingual lead is implanted but not connected (red arrow)

**Fig. 4:**
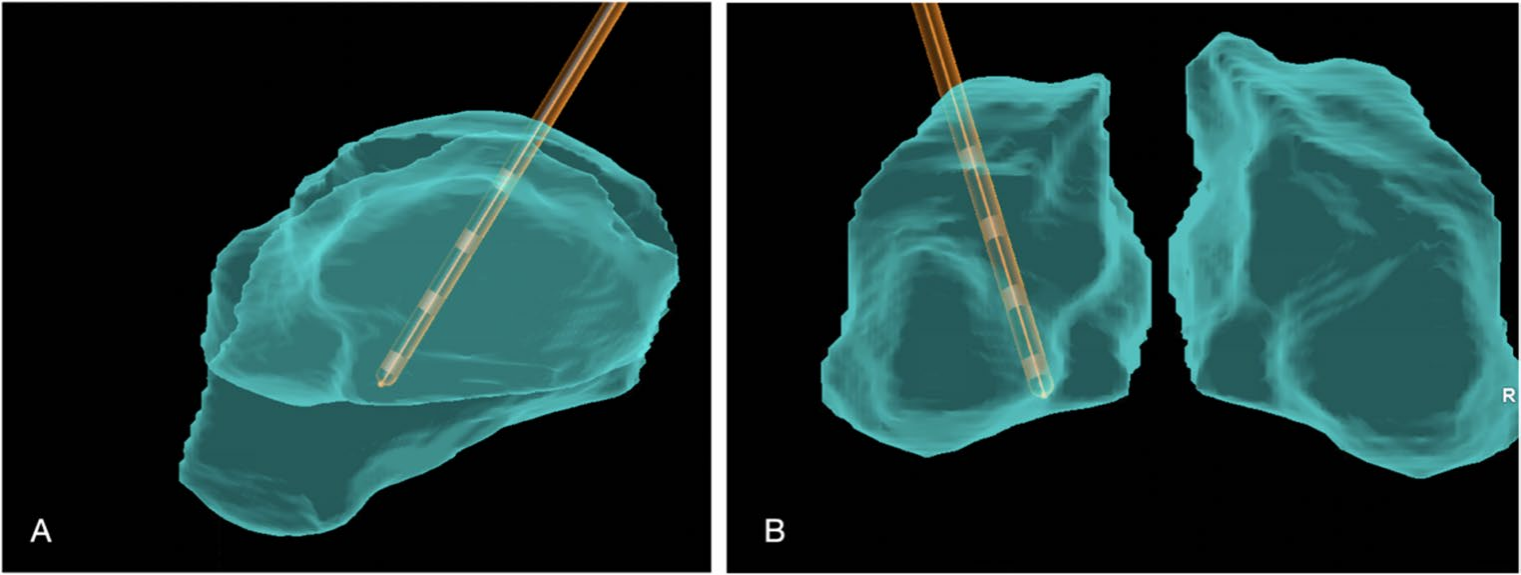
Post-operative reconstruction based on the fusion of CT scan to the MRI, using BrainLab Suite v3.4, showing the left pulvinar lead into the thalamus (in blue). The lead contacts are spaced by 3.5 mm

To further delineate the epileptogenic focus, SEEG was performed with depth electrodes targeting the following regions: hippocampal head, hippocampal body, lingual gyrus, cuneus, insula, posterior cingulate, and somatosensory cortex. Early diffuse electrographic changes were notably recorded across four contacts in the lingual gyrus electrode, rapidly spreading to the parietal region, consistent with the MEG findings in the left parietal area. Taken together, these data support a left posterior occipito-parietal network onset with the lingual gyrus as the earliest sampled node, rather than a single-gyrus focus. At our multidisciplinary epilepsy conference (epileptology, neurosurgery, clinical neurophysiology/MEG, neuroradiology, neuropsychology), consensus was that a nonlesional, near-synchronous occipito-parietal onset made focal resection or SEEG-RF thermocoagulation both high-risk for homonymous visual field deficit and low-yield in a distributed network. We therefore pursued neuromodulation, implanting responsive neurostimulation with a pulvinar target and a posterior cortical lead to engage the posterior quadrant network while preserving vision. The selected lead placements were as follows: (1) Left medial pulvinar nucleus of the thalamus, given its robust connectivity to the posterior parietal and mesial temporal cortices, (2) Left parietal region corresponding to the MEG vectors, and (3) Lingual gyrus. The implants were guided by indirect stereotactic coordinates aligned with cytoarchitectonic maps, using robot-assisted placement (ROSA). The RNS device was configured to connect the leads in the left pulvinar and parietal regions, with the third lead left unconnected due to the device’s limitation of two active leads (Figs. 3 and 4).

An initial six-week period of baseline RNS recording was undertaken, after which the device settings were optimized, and therapy was activated. The stimulation parameters were initially set as follows:

- Default Stimulation Burst 1: (+-+-) (0000) (0) 0.5 mA, 125 Hz, 160µs, 500ms, 0.5µC/cm²; Default Stimulation Burst 2: (0000) (+-+-) (0) 0.5 mA, 200 Hz, 160µs, 100ms, 0.5µC/cm².

The detection and stimulation parameters were modified every two months if necessary and the final stimulation parameters consisted of:

- Default Stimulation Burst 1: (+-+-) (0000) (0) 1.0 mA, 200 Hz, 160µs, 100ms, 1.0µC/cm²; Default Stimulation Burst 2: (0000) (+-+-) (0) 1.0 mA, 125 Hz, 160µs, 500ms, 1.0µC/cm².

**Fig. 5:**
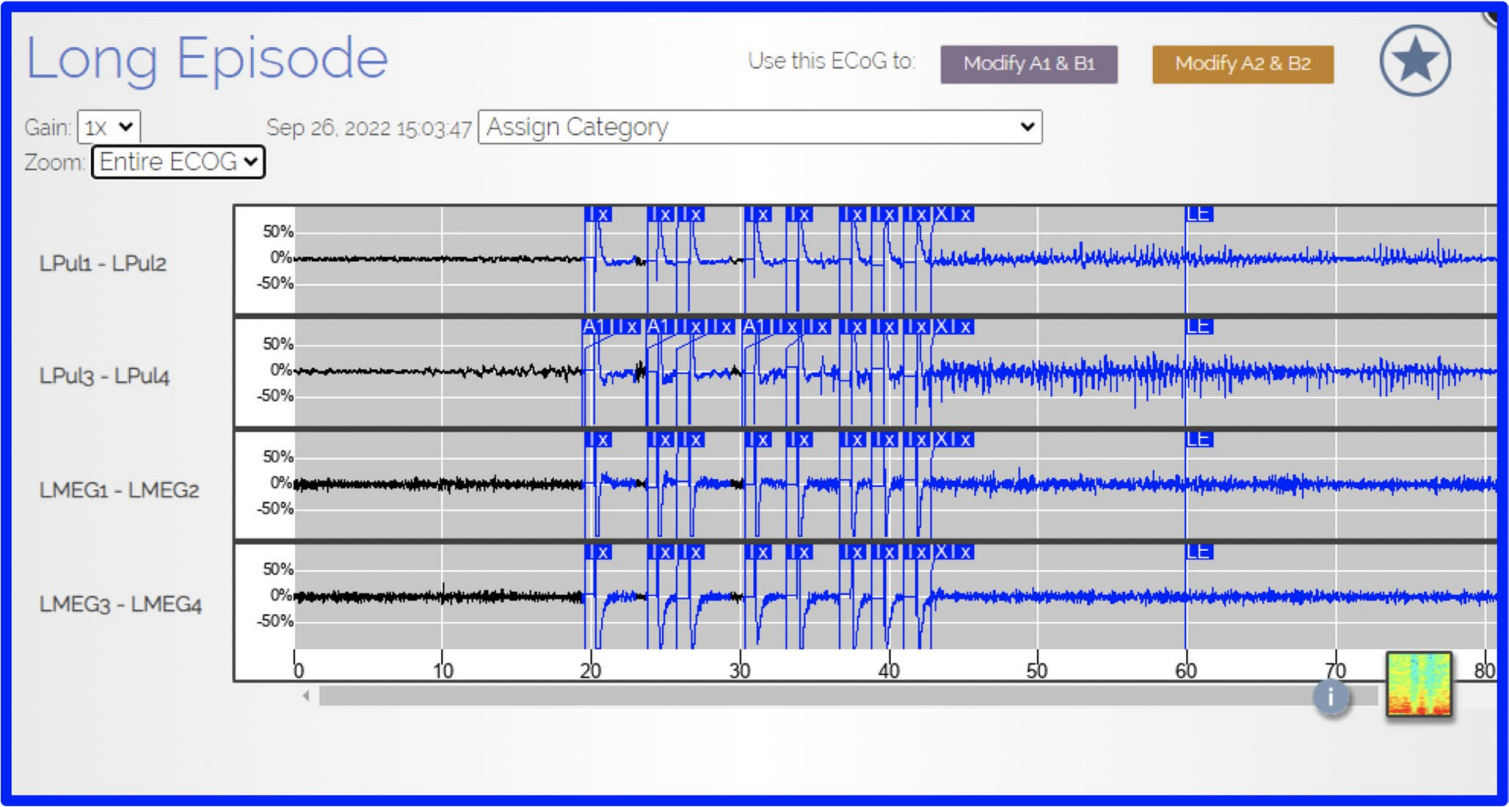
ECoG recorded from the RNS device during a long episode showing the stimulation being delivered

At baseline, the patient experienced approximately three to four impaired-awareness seizures per month and two to three focal-to-bilateral tonic–clonic seizures per year despite treatment with Keppra 3000 mg/day and Lamotrigine 600 mg/day, with prior trials of Keppra 750 mg PO BID and Topamax 150 mg PO BID without durable benefit. MEG localized posterior-quadrant activity to the left parieto-occipital region, concordant with invasive recordings. SEEG showed the earliest ictal change across four lingual-gyrus contacts with near-synchronous recruitment of the left parietal cortex, supporting a left posterior occipito-parietal network onset rather than a single-gyrus focus. At a multidisciplinary epilepsy conference (epileptology, neurosurgery, neurophysiology/MEG, neuroradiology, neuropsychology), resection/SEEG-RF was judged high-risk for homonymous visual-field deficit and low-yield; we therefore selected pulvinar-cortical responsive neurostimulation. At 18 months, seizures decreased from a baseline of 3–4/month to 0/month (100% reduction); the longest seizure-free interval was 22 months, visual fields were stable, no device-related adverse events occurred, and ASMs were reduced. Review of the ECoG library revealed multiple episodes of long interictal runs, all of which appeared to be aborted by the RNS therapy (Fig. 5).

## Discussion

The pulvinar, the largest nucleus of the primate thalamus, is characterized by its extensive afferent and efferent connections, particularly with the parieto-occipital cortex [6]. As a key associative nucleus, it functions as a major hub within the thalamocortical network, with the majority of its connections linked to various cortical regions. Classical tract-tracing techniques from the spinal cord and brainstem have not identified spinothalamic or lemniscal projections to the pulvinar [17, 19, 20]. However, advanced connectome studies have elucidated the connectivity of the dorsal lateral pulvinar with the posterior parietal cortex and the dorsal stream [21–26]. The medial pulvinar nucleus establishes direct connections with premotor, prefrontal, cingulate, and posterior parietal cortical areas, while the inferior and lateral portions are predominantly connected to the occipital cortex, closely associated with visual processing [21]. Connectome-based targeting may augment neuromodulation precision by aligning stimulation zones with patient-specific network vulnerabilities. Although our study used normative connectome data, future investigations should explore individualized tractography to refine pulvinar targeting. Compared to anterior or centromedian nuclei, the pulvinar’s posterior connectivity makes it particularly suited for posterior quadrant epilepsies [9].

Afferent inputs to the pulvinar primarily originate from the superior colliculus, targeting the inferolateral and medial regions of the nucleus. These connections are implicated in the regulation of saccadic eye movements, visual spatial attention, and higher-order visual processing [21]. The present report provides one of the few detailed clinical and anatomical results of pulvinar-responsive neurostimulation for drug-resistant epilepsy. Through this case, we were able to demonstrate the feasibility and efficacy of pulvinar RNS for long-term seizure control in often otherwise inoperable cases. Moreover, we were able to support the anatomical rationale for targeting this nucleus by leveraging connectome-based tractography. This combined clinical/anatomical approach underscores the translational potential of targeting regions based on advanced imaging tools in the aim to expand neuromodulation strategies beyond conventional thalamic sites.

The human pulvinar is increasingly recognized as a critical regulator of corticocortical communication [23] and is hypothesized to play a significant role in the modulation of seizure propagation [1, 4, 7, 27]. Acknowledging its connectivity and influence, the pulvinar has been recently proposed as a promising target for neuromodulation in epilepsy management [4, 7]. Historical use of combined lesions in the dentate and pulvinar nuclei in patients with cerebral palsy, spasticity, and complex movement disorders further underscores the pivotal role of pulvinar connections in preventing the diffuse cortical spread of abnormal signaling [28].

Emerging evidence supports the use of advanced imaging tools to refine neuromodulation targets in epilepsy, particularly within thalamic subnuclei. A recent study demonstrated the application of functional and structural connectivity to optimize responsive neurostimulation target selection, showing that pulvinar stimulation may impact large-scale networks associated with seizure propagation [29]. The pulvinar’s distributed connections are increasingly considered for cognitive and network-based modulation approaches. Additionally, graph theoretical analysis from connectomic studies highlights the pulvinar as a central hub in pathological circuits implicated in epilepsy, reinforcing its suitability for network-informed interventions [30]. Furthermore, prospective validation of pulvinar stimulation through preclinical models and normative mapping continues to support its role as a promising neuromodulation target [31]. These findings collectively emphasize the growing importance of connectome-driven strategies in defining and refining therapeutic targets for drug-resistant epilepsy.

## Limitations

The findings rely in part on connectome analysis based on normative data rather than individual patient-specific imaging, which may limit the direct clinical applicability of the tractography results. However, these data were integrated with direct clinical observations from an illustrative case, including long-term seizure outcomes after pulvinar RNS, providing translational relevance. While the preliminary case findings suggest the efficacy of pulvinar RNS in reducing seizure frequency, the study lacks a randomized controlled design, which would be necessary to establish causality and generalize the findings. The clinical sample is limited to a single case, highlighting the need for larger prospective studies to validate these observations.

## Conclusions

This study underscores the potential of the thalamic pulvinar nucleus as a novel RNS target in refractory epilepsy. Its extensive connectivity with mesial temporal, parietal, and occipital cortices suggests a key role in seizure propagation, and our illustrative case shows that pulvinar RNS can provide meaningful seizure reduction when resective surgery is not feasible. These findings remain preliminary, as the anatomical rationale relied on normative connectome data and clinical evidence is limited to a single case. Larger, multicenter studies with patient-specific connectome analyses are needed to validate the pulvinar’s utility and advance personalized neuromodulation strategies.

## Data Availability

No datasets were generated or analysed during the current study.
